# Do riparian reserves support dung beetle biodiversity and ecosystem services in oil palm-dominated tropical landscapes?

**DOI:** 10.1002/ece3.1003

**Published:** 2014-03-05

**Authors:** Claudia L Gray, Eleanor M Slade, Darren J Mann, Owen T Lewis

**Affiliations:** 1 Department of Zoology, University of OxfordOxford, U.K; 2 Department of Agricultural Sciences, University of HelsinkiHelsinki, Finland; 3 Hope Entomological Collections, Museum of Natural History, Oxford UniversityOxford, U.K

**Keywords:** Agriculture, Borneo, conservation, insect diversity, rainforest, riparian buffer

## Abstract

Agricultural expansion and intensification are major threats to global biodiversity, ecological functions, and ecosystem services. The rapid expansion of oil palm in forested tropical landscapes is of particular concern given their high biodiversity. Identifying management approaches that maintain native species and associated ecological processes within oil palm plantations is therefore a priority. Riparian reserves are strips of forest retained alongside rivers in cultivated areas, primarily for their positive hydrological impact. However, they can also support a range of forest-dependent species or ecosystem services. We surveyed communities of dung beetles and measured dung removal activity in an oil palm-dominated landscape in Sabah, Malaysian Borneo. The species richness, diversity, and functional group richness of dung beetles in riparian reserves were significantly higher than in oil palm, but lower than in adjacent logged forests. The community composition of the riparian reserves was more similar to logged forest than oil palm. Despite the pronounced differences in biodiversity, we did not find significant differences in dung removal rates among land uses. We also found no evidence that riparian reserves enhance dung removal rates within surrounding oil palm. These results contrast previous studies showing positive relationships between dung beetle species richness and dung removal in tropical forests. We found weak but significant positive relationships between riparian reserve width and dung beetle diversity, and between reserve vegetation complexity and dung beetle abundance, suggesting that these features may increase the conservation value of riparian reserves. *Synthesis and applications:* The similarity between riparian reserves and logged forest demonstrates that retaining riparian reserves increases biodiversity within oil palm landscapes. However, the lack of correlation between dung beetle community characteristics and dung removal highlights the need for further research into spatial variation in biodiversity–ecosystem function relationships and how the results of such studies are affected by methodological choices.

## Introduction

Agricultural expansion and intensification are currently among the main causes of decline in global biodiversity and ecosystem services (Phalan et al. 2013). However, large areas of agriculture will continue to be a key feature of our landscapes as the human population expands (Godfray et al. 2010). These cultivated landscapes can contribute to the persistence of biodiversity and delivery of ecosystem services, but appropriate, active management is required to achieve this (Garnett et al. 2013; Melo et al. 2013).

Successful management of biodiversity and ecological processes in tropical agricultural landscapes is especially important. Tropical landscapes are often particularly bio-diverse, highly productive for cultivation, and influence ecological functions and services on a global scale (Balmford and Whitten 2003). While primary forests are critically important for conserving tropical biodiversity and ecosystem functions (Gibson et al. 2011) and once-logged forests in Southeast Asia also have high conservation value (Edwards et al. 2011; Slade et al. 2011), focussing on these habitats alone is not sufficient. The area of land dedicated to crops or livestock is much greater than that in reserves or unmodified by humans (Ellis and Ramankutty 2008), and the landscapes surrounding protected areas may strongly influence their success (Laurance et al. 2012). Moreover, modified agricultural landscapes can also be an important habitat in their own right (Mendenhall et al. 2012).

Retaining areas of native vegetation along rivers can help maintain biodiversity and ecological functions within agricultural areas. These linear forest fragments are called riparian strips, buffer zones, stream management zones, or riparian reserves (the latter is used in Malaysia and hence in this paper). They are primarily retained because riparian forest reduces run-off into streams, improving water quality, and benefitting aquatic fauna (Sweeney et al. 2004; Mayer et al. 2007). Riparian reserves are also able to support forest-dependent communities of many terrestrial taxa, including birds, small mammals, and amphibians (Marczak et al. 2010). They are generally well protected legally and are a common feature of many agricultural landscapes (Lee et al. 2004; Barlow et al. 2010b), so offer a feasible, realistic option to improve biodiversity within cultivated areas.

Nevertheless, the ecological roles of riparian reserves remain poorly understood, particularly in Southeast Asia. The majority of existing studies on riparian reserves focus on temperate regions, and particularly on bird species (see Marczak et al. 2010 for a review). To our knowledge, there are only 15 studies in tropical regions that evaluate the ecological characteristics of existing riparian reserves, all of which focus on the neotropics or north-western Australia (Hill 1995; Laurance and Laurance 1999; de Lima and Gascon 1999; Graham and Blake 2001; Galindo-González and Sosa 2003; Harvey et al. 2006; Medina et al. 2007; Gillies and St. Clair 2008, 2010; Lees and Peres 2008; Barlow et al. 2010b; Norris and Michalski 2010; Rodríguez-Mendoza and Pineda 2010; Gillies et al. 2011; Viegas et al. 2014).

Here, we investigate the ecological impact of riparian reserves in the oil palm plantations of Sabah, Malaysian Borneo. Palm oil is now the world's primary vegetable oil, a major biofuel feedstock, and a component in many household products (Fitzherbert et al. 2008). Global annual production of palm oil more than doubled between 1970 and 2010, with over 80% of the total now produced by Malaysia and Indonesia (FAO 2014). Production in Southeast Asia is still increasing and oil palm plantations are also likely to expand in west Africa and Amazonia (Butler and Laurance 2010; Foster et al. 2011). Establishing successful conservation strategies in oil palm areas therefore has implications for landscapes across the world.

We chose dung beetles (Coleoptera: Scarabaeidae: Scarabaeinae) as a focal group to assess the conservation value of the riparian reserves. Dung beetles are described as a “high performance indicator” for tropical regions: their community metrics vary with habitat disturbance or fragmentation, they show congruency with several other taxa, and are low-cost to survey (Gardner et al. 2008; Nichols and Gardner 2011). Dung beetles also provide important ecological functions such as dung removal and bioturbation (Nichols et al. 2008), which are of wider significance to entire ecosystems.

Here, we assess whether riparian reserves support dung beetle communities and dung removal rates characteristic of larger areas of forest. We examine how the structure of riparian reserves could be managed to improve the extent to which they retain communities similar to those in logged forest. We also ask whether riparian reserves enhance the provision of dung removal services to the surrounding oil palm areas.

## Methods

### Study sites

All study sites were located within a 600 km^2^ area around and including the Stability of Altered Forest Ecosystems (SAFE) project site in Sabah, Malaysian Borneo (117.5°N, 4.6°E). The area is a mixture of twice-logged lowland dipterocarp rainforest, acacia, and oil palm plantations, in which palms were planted between 1998 and 2011. Further details are given in Ewers et al. (2011).

We selected 23 focal sites along river banks. Seven were located in logged forest (areas of continuous forest at least 500 ha in size), seven in areas of continuous oil palm with no riparian reserve adjacent to the river, and eight in areas of oil palm with a riparian reserve adjacent to the river (Fig. S1 shows these sites). One site was located in Maliau Basin primary forest reserve (70 km from the SAFE project) as a reference point, but there were no other primary forest sites near enough to allow spatial interspersion of replicate primary forest sites. As all the remaining primary forest in Sabah is already protected (Reynolds et al. 2011), evaluating the ecological characteristics of large areas of logged forest versus a network of smaller forest strips is more informative for future conservation action. All our sites were separated by at least 1.5 km, and the riparian reserve sites were at least 900 m (mean distance 3.3 km, standard deviation (SD) = 2.5 km) from the logged forest boundary. Given that dung beetle movement within a 48-h period is thought to be less than 500 m (Roslin 2000; Larsen and Forsyth 2005), it is therefore unlikely that dung beetles were drawn to the riparian reserve traps from the logged forest areas.

At each site, we set up a sampling grid of 12 points, consisting of four transects perpendicular to the river (Fig. S2). Transects were 100 m apart, with sampling points at 0 m, 50 m and 100 m from the high water line. The spacing of the grid conforms to standard methods of dung beetle sampling (Larsen and Forsyth 2005). Due to variation in width of the riparian reserves (mean 49 m, SD = 30 m, referring to forest width on one side of the river), where the riparian reserve was narrow, some points in these grids fell in the surrounding oil palm area.

### Data collection and analysis

All data were collected between the end of February and the beginning of July 2011. Seasonal changes in the lowland dipterocarp forests of Borneo are very limited (Walsh and Newbery 1999; Kumagai et al. 2005), and these months all fall in the slightly drier half of the year (Hamer et al. 2005).

All analyses were carried out in R (R Core Team 2012) using the packages vegan (Oksanen et al. 2013), lme4 (Bates et al. 2012) and nlme (Pinheiro et al. 2013).

#### Dung beetle community and land use

Dung beetles were collected using pitfall traps baited with 25 g of human dung. Human dung attracts a wide variety of species (Davis et al. 2001; Larsen et al. 2006) and is recommended as a standardized bait in tropical forests (Marsh et al. 2013). Each trap consisted of a plastic cup (8-cm top diameter, 5.5-cm bottom diameter, and 12.5-cm depth) half-filled with a solution of water, detergent, and salt. The traps were protected from the rain with a cover and collected after 48 h. The order of sites was randomized, and traps were set at no more than two sites in each 48-h period.

We could not obtain sufficient human dung to supply both the traps and the dung piles, so we used cattle dung for the dung removal experiment. Preliminary work in similar forest sites in Sabah shows that large cattle dung baits attract a similar species composition to smaller human dung baits, with the exception of some carrion feeding species found in higher abundances in human dung (Slade et al. 2011, E. Slade and D. Mann, unpubl. data). To compare species and dung removal results, we removed data on these carrion feeding species (*n* = 13, highlighted in Table S1) from all analyses apart from those testing for the effect of riparian reserve structural features on the entire dung beetle community.

For each sampling point (trap), we calculated dung beetle abundance, the number of functional groups present (using classifications based on diurnal vs. nocturnal activity, body length, and method of dung removal after Slade et al. (2007)), *α* diversity (Shannon index), and total biomass. We weighed beetles from 24 species taken from across the whole range of body sizes (between 7 and 51 individuals per species, average = 27, SD = 8) and used a polynomial regression to estimate biomass for the remaining species (Log_10_(mass) = −1.64 + 5.61*Log_10_(length) − 4.39*Log_10_(length)^2^ + 1.99*Log_10_(length)^3^, *R*^2^ = 0.982).

For each site, we calculated *β* diversity (mean Sørensen's similarity index) and species richness (using coverage-based rarefaction methods (Chao and Jost 2012) through the iNEXT online software (Hsieh et al. 2013). Coverage-based methods of rarefaction provide a more informative comparison of richness among multiple samples than individual or sample-based methods of rarefaction as the ratio of species richness is not compressed (Chao and Jost 2012). Rarefied species richness could not be calculated at the trap level due to four traps having only one or two beetles.

Wherever possible, we retained data at the highest spatial resolution (trap level) for analyses. For response variables where this was the case (abundance, functional group richness, diversity, and biomass), we analyzed the effect of land use (logged forest, riparian reserve, or oil palm) with generalized linear mixed models, using transect nested within site as a random factor. Where response variables could only be calculated at the site level (*β* diversity and rarefied species richness), we analyzed the effect of land use with a generalized least squares model. For all models, appropriate error distributions were specified and transformations or weight structures (varIdent function as described by Zuur et al. (2009)) applied where necessary. For all analyses testing for an effect of land use, we excluded data from points that fell outside of the forest strip at the narrowest riparian reserve sites so that we were carrying out a true test for differences between the three land uses. Some traps were lost due to flooding or other disturbances, so data were only obtained from 201 traps in total (82 from logged forest sites, 43 from inside riparian reserves, and 76 from oil palm sites).

Differences in community composition across land uses were explored using de-trended correspondence analysis (DCA, vegan function “decorana”), which performs well as an ordination method for displaying similarity of tropical insect communities along an environmental gradient (Brehm and Fiedler 2004). We tested for significant differences in community composition using a permutational analysis of variance (vegan function “adonis”) with 999 permutations and site as a grouping variable.

To determine whether the relative abundance of the functional groups differed between logged forest and riparian reserves, we ran a mixed model with abundance as a response variable and functional group, land use, and their interaction term as predictors.

#### Dung removal and land use

To record dung removal activity, uniform pats of 700 g of cow dung were set out at each sampling point (*n* = 12 at each site) and collected after 24 h. Large herbivores, such as the tembadau or wild cow (*Bos javanicus* d'Alton), Asian elephant (*Elephus maximus* L.), and bearded pig (*Sus barbatus* Müller), occur within the study area so the experimental dung pats resemble those occurring naturally. Dung removal experiments were carried out at least 1 month after pitfalls traps were collected, in order to avoid interference but also remain close enough for dung beetles assemblages to be similar (Slade et al. 2011). The order in which sites were visited was randomized. The dung was frozen for a minimum of 24 h before the experiment to kill any invertebrates already present. Data on mass loss were corrected for evaporation using estimates from three evaporation controls set at each site. For the controls, the cow dung was placed in a flat-bottomed sieve with mosquito netting sealed around the top (both 1-mm mesh), to prevent entry of any dung beetles.

The effect of land use on the mass of dung removed was analyzed with a general linear mixed model, with transect nested in site as random factors and a log transformation for the response variable. As with the data for beetle communities, for the riparian reserve sites, we only used data from within the forest strips (total *n* = 212: 84 from logged forest sites, 44 from within riparian reserve vegetation, 84 from oil palm sites).

We assessed whether the relationship between dung beetle community characteristics and dung removal was consistent across all land use types with a generalized linear model including land use, rarefied species richness (correlated with diversity *R*^2^ = 0.6, *P* = 0.004), biomass (correlated with abundance, *R*^2^ = 0.7, *P* = 0.0002), functional group richness, and all two-way interactions. As this analysis included coverage-based rarefied species richness all other data were averaged to the site level.

#### Dung beetle community structure and riparian reserve characteristics

To analyse the effect of riparian reserve width and vegetation complexity on the dung beetle community, we included data on all dung beetle species (both carrion and dung feeders). In order to test whether increasing the proportion of area in the riparian zone left as native vegetation impacts the dung beetle community, we included all the points in the sampling grid at each riparian reserve site (*n* = 95). Data were combined for each transect as this was the resolution at which riparian forest width could be measured (using GIS software [ArcMap version 10.1, ESRI, Redlands, CA]). A similar approach was used by Viegas et al. (2014) to test whether reserve width affects dung beetle communities in the Amazon.

To assess the vegetation structure at each sampling point, we measured humus depth, canopy density (using a spherical densitometer), and basal area (using the angle point method (Bitterlich 1984)). We estimated the height of the tallest tree to the nearest 5 m using a ruler held at arm's length and a known reference height at the base of the tree. We scored the understorey vegetation density (below 2 m) and midstorey vegetation density (between 2 m and 5 m) on an ordinal scale of sparse (fewer than 20 stems or branches), medium (20–60 stems or branches), and dense (few patches of light and 60–100 + stems or branches). To obtain one numerical index summarizing the greatest variation in these data, we ran a metric scaling analysis on all these measurements. The first axis was positively correlated with canopy density, tree height, humus depth, basal area, and midstorey density. Because this output is therefore capturing variation in the three-dimensional structure of the habitats, we refer to it as a vegetation complexity index.

We analyzed the effect of vegetation complexity on dung beetle abundance, biomass, diversity, functional group richness, and species richness using only data from sampling points falling within the riparian reserve forest. To test for any effects of reserve width or vegetation complexity, we used generalized linear mixed models, with site as a random factor and specified error families where appropriate.

#### Provisioning of dung removal services by riparian reserves

We analyzed the effect of riparian reserves on dung removal rates in the surrounding oil palm area in two ways. First, we compared the dung removed in oil palm adjacent to a riparian reserve (i.e., from sampling points at riparian reserve sites that fell outside the riparian forest, *n* = 52) and in oil palm without an adjacent riparian reserve (and also at least 50 m from the river bank, *n* = 56). Second, using only the data from sampling points in oil palm adjacent to a riparian reserve, we analyzed the effect of distance from the riparian reserve boundary on the mass of dung removed. We used a generalized linear mixed model with the presence/absence of riparian reserve or distance from the reserve boundary as a fixed factor for the two analyses, respectively. In both cases, we specified transect nested within site as a random factor and applied log transformations to meet model assumptions.

## Results

### Dung beetle community structure and land use

In total, we identified 73 species from 9135 dung beetles (Table S1). The iNEXT software estimate for species coverage of the raw data was greater than 0.91 (>91% of species present were recorded) at all sites, and all species richness curves were approaching the asymptote (Fig. S3), indicating that we had sampled the community thoroughly.

**Figure 1:**
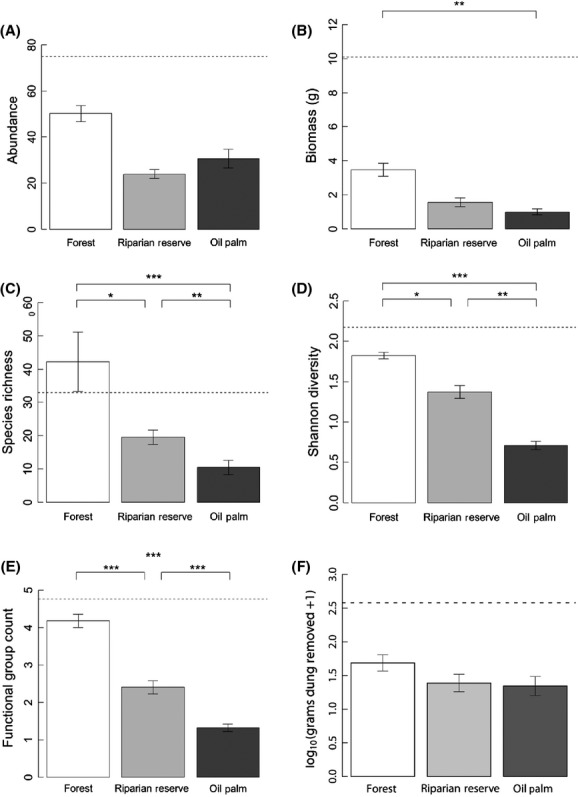
Effect of land cover on (A) dung beetle abundance, (B) biomass, (C) coverage-based rarefied species richness, (D) diversity (Shannon index) (E) functional group richness and (F) dung removal. All panels show means and standard errors. The dotted lines indicate values for the one primary forest reference site (for visual comparison only; the data were not included in the analysis). Stars denote significant differences between groups based on model contrasts (*P < 0.05, **P < 0.01, ***P < 0.001).

We found a significant effect of land use on dung beetle biomass and a weakly significant effect of land use on abundance (Table 1). The biomass in riparian reserves was intermediate between oil palm and logged forest, but not significantly different from either (Fig. 1B).

**Table 1:** Effects of land cover and habitat characteristics on dung beetle community metrics and dung removal using data at the trap level (or pooled to transect level for analyses with width as a fixed factor). Test statistics given for comparison of model specified against the null model (response – 1).

Model	*χ*^2^	df	*P*
*Dung beetle community response to land use*			
Abundance ∼ land cover	5.9	2	0.051
Biomass ∼ land cover	7.9	2	0.019*
Shannon diversity ∼ land cover	22.7	2	<0.0001***
Functional group count ∼ land cover	28.8	2	<0.0001***
*Dung beetle community response to reserve width*			
Abundance ∼ riparian reserve width	0.62	1	0.43
Biomass ∼ riparian reserve width	0.05	1	0.82
Species richness ∼ riparian reserve width	3.69	1	0.055
Shannon diversity ∼ riparian reserve width	5.45	1	<0.02*
Functional group richness ∼ riparian reserve width	1.15	1	0.28
*Dung beetle community response to vegetation complexity*			
Abundance ∼ vegetation complexity	5.95	1	0.015*
Biomass ∼ vegetation complexity	0.54	1	0.46
Species richness ∼ vegetation complexity	0.3	1	0.58
Shannon diversity ∼ vegetation complexity	0.0004	1	0.98
Functional group richness ∼ vegetation complexity	0.0005	1	0.98
*Dung removal function*			
Dung removed ∼ land cover	4.6	2	0.10
Dung removed in oil palm ∼ the presence/absence of riparian reserve	0.58	1	0.45
Dung removed in oil palm ∼ distance to riparian reserve boundary	2.11	1	0.15
*Riparian reserve features*			
Vegetation complexity ∼ riparian reserve width	1.8	1	0.18

Significant differences between the model described and the null model

* *P* < 0.05;

** *P* < 0.01;

*** *P* < 0.001

Species richness (Table 2), diversity, and functional group richness (Table 1) also varied significantly with land use. Riparian reserve species richness (Fig. 1C), diversity (Fig. 1D), and functional group richness (Fig. 1E) were significantly lower than in logged forest and higher than in oil palm. We found no difference in within-site *β* diversity (mean Sørensen's similarity index) among the different land use types (Table 2).

**Table 2:** Effects of land cover and habitat characteristics on dung beetle community metrics and dung removal on response variables that could only be calculated at site level.

Model	*F*	df	*P*
Species richness ∼ land cover	16.9	2,19	<0.0001***
*β* diversity ∼ land cover	1.9	2,19	0.18
Dung removed ∼ sp.rich*land.cov + biomass*land.cov + f.rich*land.cov	0.8	11,10	0.67
Dung removed ∼ sp.rich + biomass + f.rich	1.7	3,18	0.2

sp.rich, species richness; land.cov, land cover; f.rich, functional group richness.

Significant differences between the model described and the null model

* *P* < 0.05;

** *P* < 0.01;

*** *P* < 0.001).

Three of the seven functional groups were missing completely from oil palm sites: small nocturnal tunellers, small diurnal rollers, and large nocturnal rollers. In contrast, all functional groups were found in at least one of the riparian reserves sites. However, we found a significant interaction between land cover and functional group on dung beetle abundance (*χ*^2^ = 59.8, df = 6, *P* < 0.0001). This indicates that functional groups vary in the extent to which they are negatively impacted by the conversion from logged forest to riparian reserve (Table 3). The most negatively impacted functional groups were the large diurnal tunellers and large diurnal rollers.

**Table 3:** Model output of generalized linear mixed model (dung beetle abundance – land cover * functional group), showing parameter estimates and standard error for the percentage decline in abundance of each functional group in riparian reserve sites relative to logged forest sites.

Functional group	No. species in group	Estimate % decline	Standard error % decline	*P*
Large diurnal tunellers	1	89.48	17.70	0.0002***
Large diurnal rollers	2	80.19	15.78	0.0002***
Small diurnal rollers	1	71.92	12.74	0.0001***
Small diurnal tunellers	21	18.65	5.60	0.0054**
Large nocturnal tunellers	4	32.96	18.64	0.3
Small nocturnal tunellers	3	95.32	71.8	0.2
Large nocturnal rollers	1	86.48	207.1	0.7

Significant denotes

* *P* < 0.05;

** *(P* < 0.01;

*** *P* < 0.001).

**Figure 2:**
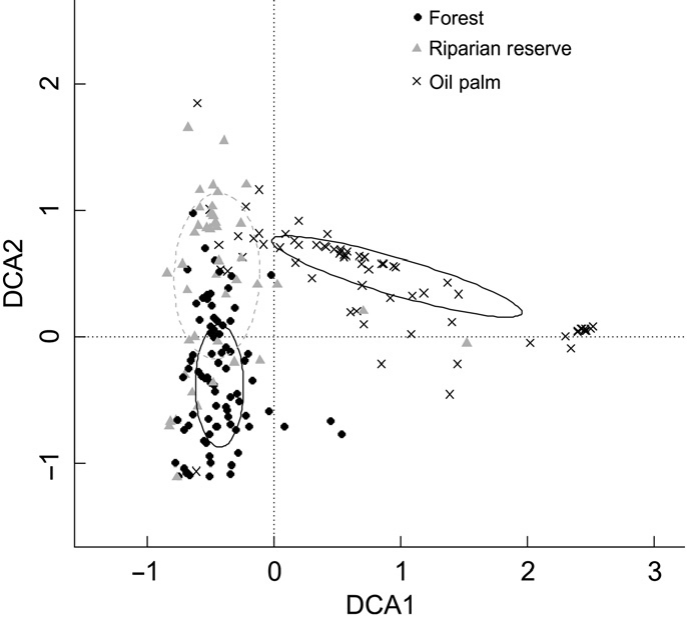
Detrended correspondence analysis (DCA) plot indicating that riparian reserve community composition is more similar to forest than oil palm. Ellipses show standard deviation around the mean for each land use.

The community composition of the riparian reserves was more similar to logged forest than oil palm, although a distinct difference in the communities of the reserves and larger forested areas remains (*F*_1,193_ = 21.4, *P* = 0.001, Fig. 2).

The single primary forest reference site (not included in the analyses above) had much higher mean dung beetle abundance (145% of logged forest), biomass (319% of logged forest), diversity (115% logged forest), and functional group richness (114% logged forest) than all other land use types (Fig. 1). However, the species richness of the reference primary forest site fell within the range of the logged forest sites (Fig. 1C).

### Dung removal and land use

The proportion of dung removed across all sites was low (mean = 0.1, SD = 0.14). There was no significant relationship between dung removal and land cover, species richness, diversity, functional group richness, or any of the two-way interactions (Table 2).

### Dung beetle community and riparian reserve characteristics

There was no significant relationship between riparian reserve width and vegetation complexity (Table 1).

**Figure 3:**
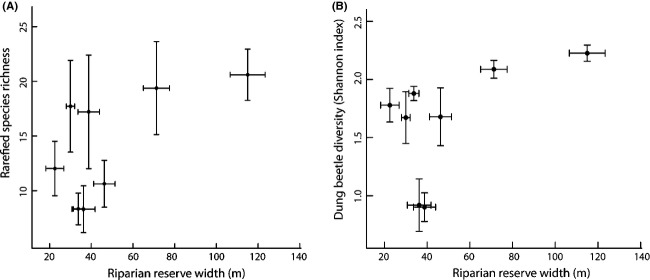
Relationship between riparian reserve width and (A) rarefied species richness and (B) diversity (Shannon index). Plots show mean ± standard error for each replicate site.

We found no evidence of a relationship between reserve width and dung beetle abundance, biomass, or functional group richness (Table 1). However, there was a significant positive relationship between width and diversity and a weakly significant positive relationship between riparian reserve width and dung beetle rarefied species richness (Shannon index, Table 1, Fig. 3).

We found a positive relationship between the vegetation complexity of the riparian reserve forest and beetle abundance (Table 1). However, we found no significant effects of vegetation complexity on biomass, species richness, diversity, or functional group richness (Table 1).

### Provisioning of dung removal services by riparian reserves

Dung removal did not differ significantly between oil palm with and without riparian reserves, and we found no significant effect of distance from the riparian reserve boundary on the mass of dung removed (Table 1).

## Discussion

The rapid expansion of oil palm plantations throughout the tropics threatens many forest species. While large protected areas will undoubtedly remain the priority for conservation in these areas, riparian reserves are a potential opportunity to increase the biodiversity retained within agricultural areas. Our data show that, compared with areas of oil palm, riparian reserves of at least 30 m width (on each side of the river) support dung beetle communities more similar to those in adjacent areas of logged forest. We found some effects of reserve width and vegetation complexity on the diversity and abundance of dung beetles in riparian vegetation, suggesting that these structural features may also make a limited contribution the biodiversity benefits of the riparian reserves.

Previous studies on dung beetle communities and dung removal function in Bornean rainforests have shown a positive relationship between dung beetle species or functional group richness and dung removal function (Slade et al. 2007, 2011). Our data suggest that this relationship may not hold in riparian zones and that the presence of a riparian reserve does not increase dung removal in surrounding areas of oil palm.

### Dung beetle community structure and land use

We found that dung beetle communities in oil palm plantations had lower biomass, species richness, diversity, and functional group richness than larger areas of forest. Similar studies in Borneo have also found that dung beetle species richness and diversity declines with logging and conversion to plantations (Davis et al. 2001; Edwards et al. 2013). In contrast to these studies, we did not find a decline in dung beetle abundance across the land use gradient, but this may be because we did not make a comparison with primary forest sites.

The species richness, functional group richness, diversity, and overall community composition of dung beetle communities within riparian reserves were more similar to forest than oil palm. These results provide strong evidence that protecting riparian reserves retains biodiversity within oil palm landscapes, when compared to plantations where oil palm is planted up to the river bank. Riparian reserves are not, however, an adequate substitute for large areas of logged or primary forest. In addition, further work is needed to establish whether dung beetles are able to maintain viable, self-supporting populations within riparian reserves, rather than comprising transient visitors or sink populations (Barlow et al. 2010a).

The functional diversity of the oil palm was lower than both the logged forest and riparian reserves. Rollers and nocturnal species were particularly negatively affected by conversion to oil palm. The greater sensitivity of these groups after conversion to oil palm was also reported by Edwards et al. (2013) and may be connected to limited temperature tolerance. The riparian reserve network retained all functional groups, but each site tended to support fewer functional groups than logged forest sites. The drop in average functional group richness between forest and riparian reserves was due to the loss of diurnal species (both small and large, rollers and tunellers), which may be due to a decline in diurnal mammal species (Andresen and Laurance 2007). These results suggest that the isolation of forest strips results in different trait-dependent responses compared to conversion to oil palm. Our findings contrast the global study carried out by Nichols et al. (2013a), who found that for the afro-eurasian tropics nocturnal species are more affected by forest modification, and diurnal species are more negatively affected by conversion to plantation. However, Nichols et al. (2013a) were comparing all forest modification to a primary forest baseline, whereas we are comparing oil palm and riparian reserves to a logged forest baseline, which may explain this discrepancy.

As well as supporting dung beetle species that would not survive if oil palm were planted along river banks, riparian reserves are likely to benefit a range of other taxa. In Borneo, riverine forest corridors are recognized as important habitat for some mammalian species, including the orang-utan, proboscis monkey, and pygmy elephant (Venkataraman et al. 2009), but little research has been carried out on the importance of riparian reserves for many other groups in this region. Riparian reserves in the neotropics support communities of birds, amphibians, and small mammals found in undisturbed forest (de Lima and Gascon 1999; Lees and Peres 2008) and also facilitate movement of forest specialists through agricultural land (Gillies and St. Clair 2008). Alongside the hydrological benefit of riparian reserves, their role in conserving terrestrial species should be more widely recognized by sustainable management guidelines. This is especially the case in Sabah where all the remaining primary forest is already protected and increasing conservation in cultivated landscapes is arguably the highest priority (Reynolds et al. 2011).

### Dung removal and land use

Despite the significant differences among beetle communities in different land uses, we did not detect a significant effect of land cover on dung removal rates over 24 h, nor any significant relationship between dung removal and species richness, biomass or functional group richness. This contrasts a number of studies that show strong positive correlations between dung beetle species richness or functional group richness and dung removal rates (Slade et al. 2011; Braga et al. 2013; Gollan et al. 2013), and also with evidence that dung removal rates in Amazonian riparian reserves are higher than in surrounding pasture (Norris and Michalski 2010).

There are several possible explanations for these results. First, dung removal rates were low (e.g., compared with those in primary forest nearby; Fig. 1F), and it is possible that a difference in removal would be seen if dung pats were left out for longer, and a greater proportion of mass was removed. Secondly, it is possible that differences in the communities attending the two bait types diminish our ability to detect correlations between biodiversity and function; Nichols et al. (2013b) discuss how dissimilarities between the response of dung beetles communities and dung burial rates to human impact in the Amazon may be an artifact of surveying the community and function at separate times with different baits. However, correlations have previously been detected using different baits within similar forests in Borneo (Slade et al. 2011). Thirdly, it is possible that there is spatial variation in the relationship between dung beetle community composition and dung removal. Because the mortality of dung beetle larvae may increase with soil moisture content (Sowig 1995), dung beetles may not build nests (bury dung) near rivers even though they come to baits to feed. The positive relationship between species richness and dung removal may therefore break down in riparian zones, but additional data on how dung removal and soil moisture vary is needed to confirm this. Therefore, while there may be local and regional variation in biodiversity–ecosystem function relationships within tropical forests, the extent to which these relationships are affected by sampling methodology needs to be further resolved.

The lower functional group richness in riparian reserves compared with forest may affect important ecological processes inside the reserves. In particular, roller species are less abundant in the reserves. As these species roll dung balls, often containing seeds, horizontally away from the dung pat they can potentially reduce the negative effects of seed clumping and seedling competition (Lawson et al. 2012). It is therefore possible that germination and dispersal dynamics of plant species in the reserves are impaired relative to logged forest. Other processes such as soil bioturbation, soil fertilization, and parasite suppression that are mediated by dung beetles (Nichols et al. 2008) may also be reduced in the riparian reserves as a result of the decline in some functional groups.

### Dung beetle community structure and riparian reserve characteristics

Our results suggest that the width and vegetation complexity of riparian reserves may have a positive impact on dung beetle diversity and abundance, respectively. Although these relationships are weak, these findings are of direct relevance to management and policy specifications. Legal requirements for the protection of riparian forest exist in a number of countries (Sabah Water Resources Enactment, 1998; Barlow et al. 2010b; Marczak et al. 2010; FAO, 2014), and riparian reserves are also included in the criteria for certification of sustainable palm oil production (e.g., by the Round Table on Sustainable Palm Oil (Criterion 4.4 (RSPO 2013)). However, very little ecological information has influenced the details of these guidelines (Barlow et al. 2010b; Ewers et al. 2011). Our findings highlight the need for further research to clarify the importance of the structural features of riparian reserves.

### Provisioning of dung removal services by riparian reserves

Grazing of cattle underneath oil palms is expanding in Malaysia (Latif and Mamat 2002), and the requirement for dung removal services within these landscapes is likely to increase. However, our results suggest that retaining riparian reserves within oil palm plantations may not contribute to an increase in dung removal services within surrounding oil palm.

## Conclusions

Overall, it is evident that riparian reserves can contribute toward the conservation of dung beetle communities that are threatened by the expansion of oil palm, but that the extent to which they support dung removal activity and other terrestrial ecosystem services requires further study over greater spatial and temporal scales. Nevertheless, the results presented here indicate that riparian reserves should be more widely recognized as a conservation strategy for terrestrial biodiversity. We must emphasize that we do not recommend riparian reserves within oil palm plantations as an alternative to protecting large areas of primary or secondary forest. On the contrary, we feel that as an addition to such protection, riparian reserves should be more widely recognized as a promising opportunity for conservation in tropical agricultural landscapes.

## Supporting Information

**Figure S1.** Map of sites used in this study. **Figure S2.** Diagram of sampling design. **Figure S3.** Species accumulation curves for the different land uses. **Table S1.** Species list and taxonomic reference for all dung beetles identified in this study.
