# Supplementary material for: Do riparian reserves support dung beetle biodiversity and ecosystem services in oil palm-dominated tropical landscapes?
Source: Ecol Evol. 2014 Mar 5;4(7):1049–60. doi: 10.1002/ece3.1003 (PMC3997321; doi:10.1002/ece3.1003)
Supplement: Supplementary file 1 — Figure S1. Map of sites used in this study. Figure S2. Diagram of sampling design. Figure S3. Species accumulation curves for the different land uses. Table S1. Species list and taxonomic reference for all dung beetles identified in this study. [file ece30004-1049-sd1.docx]

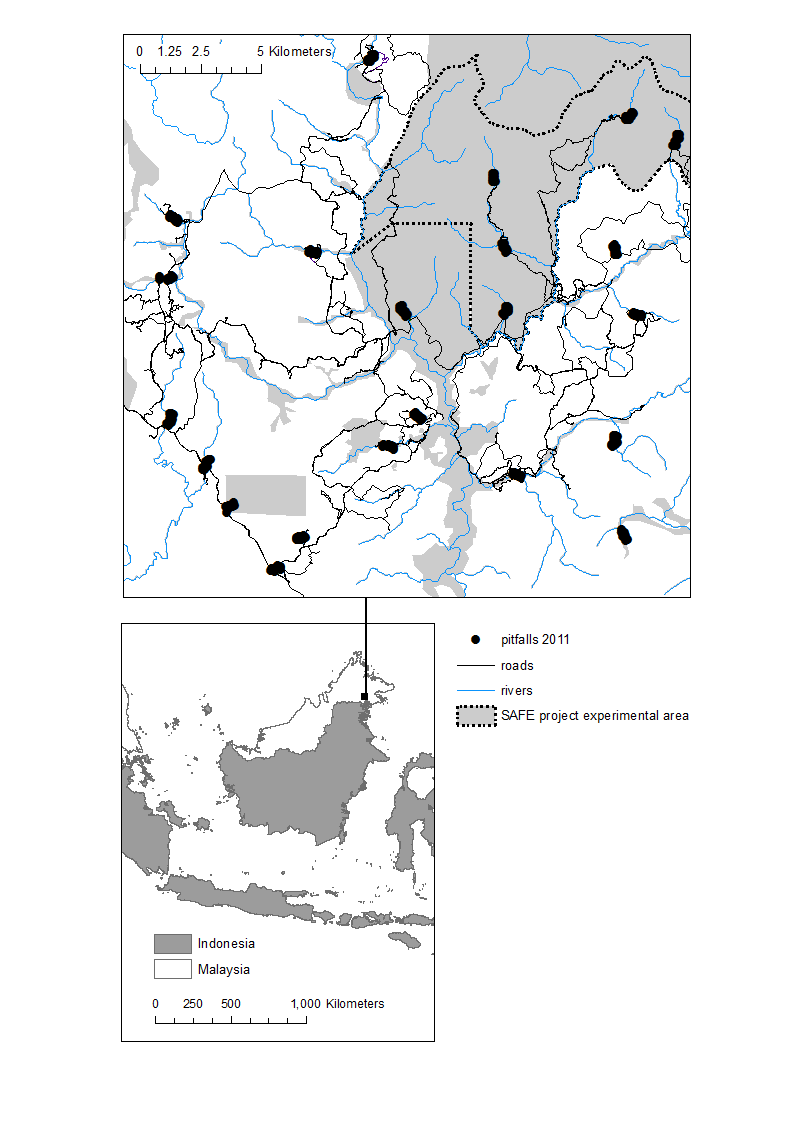


Figure S1. A map of the field sites used in this study. The dotted black line gives the boundary of the SAFE experimental area. The solid grey shaded area is the remaining logged forest, and the white area is the matrix of plantations. The sampling points are shown by black circles. The lower panel gives the location of the study area within Sabah, Northern Borneo.

Table S1. Full species names and taxonomic reference for the dung beetles caught and identified. Those in bold are primarily fruit or carrion feeders and were removed from all analyses on the effect of land cover so comparisons could be made to dung removal data.

Species name Taxonomic reference

*Caccobius (Caccobius) bawangensis*  Ochi, Kon & Kikuta, 1997

*Caccobius (Caccobius) binodulus* Harold, 1877

*Catharsius dayacus* Lansberge, 1886

*Catharsius renaudpauliani*  Ochi & Kon, 1996

*Copris (Copris) agnus*  Lansberge, 1875

*Copris (Paracopris) ramioceps* Gillet, 1921

*Copris (Copris) sinicus* Hope, 1842

*Microcopris doriae* Harold, 1877

*Microcopris hidakai* Ochi & Kon, 1996

***Ocicanthon masumotoi* Ochi & Arayi, 1996**

***Ocicanthon dytiscoides* Boucomont, 1914**

***Ocicanthon danum* Krikken & Huijbregts, 2007**

***Ocicanthon woroae* Ochi, Ueda & Kon, 2006**

*Oniticellus tessellatus*  Harold, 1879

*Onthophagus (Micronthophagus) aff. araya* Ochi & Kon, 2007

*Onthophagus (Sinonophagus) aff. angastatus sp. 1* Boucomont, 1914

*Onthophagus (Sinonophagus) aff. angastatus sp. 2* Boucomont, 1914

*Onthophagus (Onthophagus) aphodioides* Lansberge, 1883

***Onthophagus (Parascatonomus) aurifex*  Harold, 1877**

*Onthophagus (Onthophagus) batillifer* Harold, 1875

*Onthophagus (Onthophagus) borneensis* Harold, 1877

***Onthophagus (Parascatonomus) brendeli* Ochi, Kon & Barclay, 2009**

*Onthophagus (Gibbonthophagus) cervicapra* Boucomont, 1914

*Onthophagus (Pseudophanaeomorphus) chandrai*  Ochi, 2007

*Onthophagus (Onthophagus) deflexicolis* Harold, 1877

*Onthophagus (Onthophagiellus) aff. deliensis* Lansberge, 1885

***Onthophagus (Parascatonomus) dux* Sharp, 1875**

*Onthophagus (Onthophagiellus) aff. falculatus* Boucomont, 1914

*Onthophagus (Gibbonthophagus) fujii*  Ochi & Kon, 1995

*Onthophagus (Onthophagiellus) aff. hidakai* Ochi & Kon, 1995

*Onthophagus (Onthophagus) incisus* Harold, 1877

*Onthophagus (Indachorius) aff. cheyi* Ochi & Kon, 2006

*Onthophagus (Indachorius) danumensis* Ochi, Kon & Barclay, 2009

*Onthophagus (Indachorius) aff. woroae* Ochi & Kon, 2006

*Onthophagus (Pseudophanaeomorphus) johkii* Ochi & Kon, 1994

*Onthophagus (Onthophagiellus) aff. kawaharai* Ochi & Kon, 2007

*Onthophagus (Serrophorus) laevis* Harold, 1880

*Onthophagus (Serrophorus) muelleri* Lansberge, 1883

*Onthophagus (Onthophagus) aff. borneensis* Harold, 1877

*Onthophagus (Gibbonthophagus) nigriobscurior*  Ochi, Kon & Tsubaki, 2009

*Onthophagus (Onthophagus) obscurior* Boucomont, 1914

*Onthophagus (Onthophagus) ochromerus* Harold, 1877

*Onthophagus (Pseudophanaeomorphus) aff. phanaeides* Frey, 1956

*Onthophagus (Onthophagiellus) aff. tridentitibialus* Ochi & Kon, 2008

*Onthophagus (Onthophagus) pacificus agg.* Lansberge, 1895

*Onthophagus (Onthophagus) pastillatus*  Boucomont, 1919

*Onthophagus (Onthophagus) pavidus* Harold, 1977

*Onthophagus (Onthophagus) simboroni* Ochi & Kon, 2006

*Onthophagus (Pseudophanaeomorphus) quasijohkii* Ochi & Kon, 2005

*Onthophagus (Sinonthophagus) rorarius*  Harold, 1877

***Onthophagus (Parascatonomus) rudis* Sharp, 1875**

*Onthophagus (Onthophagus) rugicollis*  Harold, 1880

*Onthophagus (Onthophagus) aff. rutilans* Sharp, 1875

***Onthophagus (Parascatonomus) sarawacus*  Harold, 1887**

***Onthophagus (Parascatonomus) semiaurreus* Lansberge, 1883**

***Onthophagus (Parascatonomus) semicupreus* Harold, 1877**

*Onthophagus (Indachorius) aff. semidanumensis* Ochi, Kon & Barclay, 2009

*Onthophagus (Gibbonthophagus) taeniatus* Boucomont, 1914

*Onthophagus (Paraphaenaemorphus) trituber*  Wiedemann, 1823

*Onthophagus vethi* Krikken, 1977

*Onthophagus (Onthophagus) vulpes* Harold, 1877

*Onthophagus (Onthophagus) waterstradti* Boucomont, 1914

*Onthophagus (Onthophagus) sp. A*

*Onthophagus (Onthophagus) sp. LGD*

***Panelus sp. 1***

***Panelus sp. 2***

*Paragymnopleurus maurus* Sharp, 1875

*Paragymnopleurus sparsus* Sharp, 1875

*Paragymnopleurus striatus* Sharp, 1875

*Proagoderus watanebei*  Ochi & Kon, 2002

*Synapsis ritsemae* Lansberge, 1874

*Sysyphus thoracicus* Sharp, 1875

*Yvescambefortius sarawakus*  Gillet, 1926


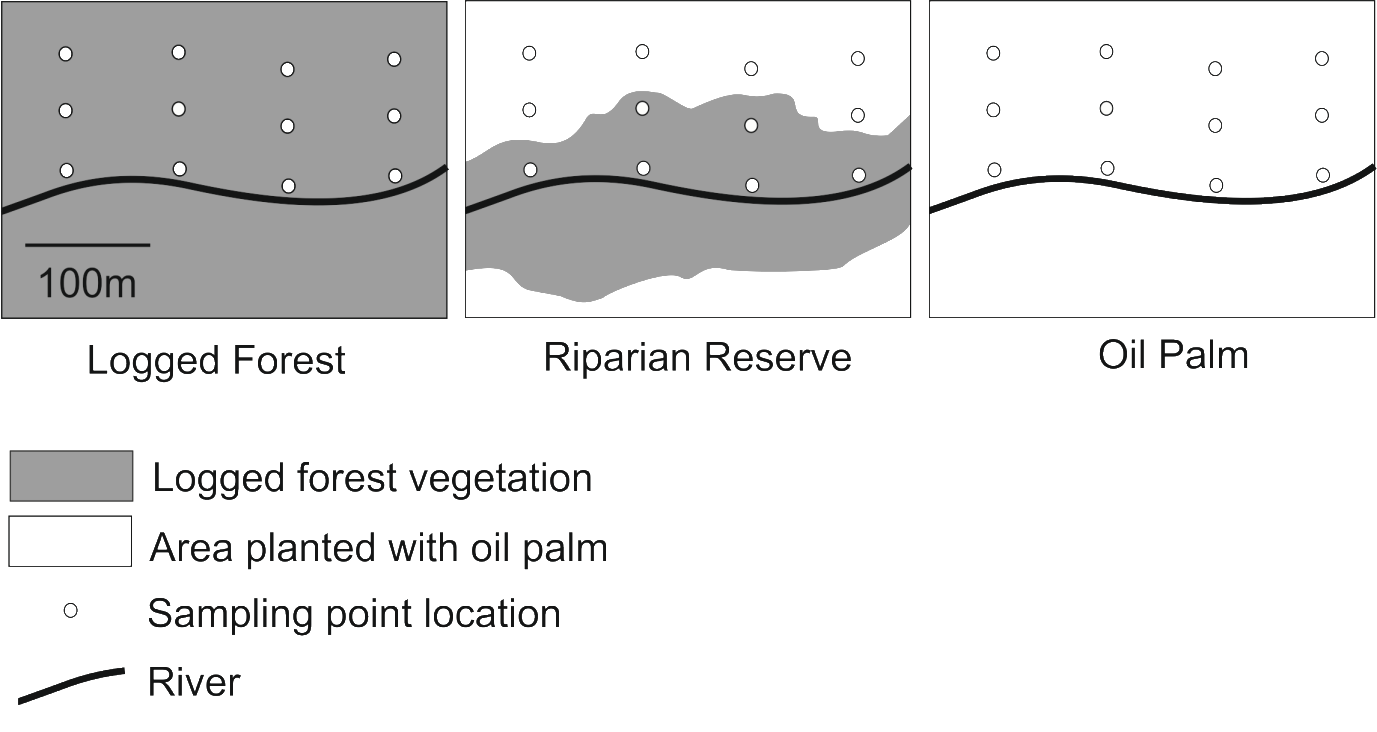


Fig. S2 Arrangement of sampling points in riparian zones within each land use. Distribution of points follows the standard approach for dung beetle sampling, and was kept constant across all land uses as the density of baited traps might affect trap attractiveness. Data from all the forest points, all the oil palm points, and the points within the riparian forest were used to assess differences in dung beetle community and dung removal function across land uses. Data from within the riparian reserves only was used to test for effects of riparian reserve vegetation complexity on community metrics. The data from all points at the riparian reserve sites was used to assess the effects of increasing the area of non-crop vegetation (i.e. increasing riparian reserve width) within the riparian zone of the plantations.


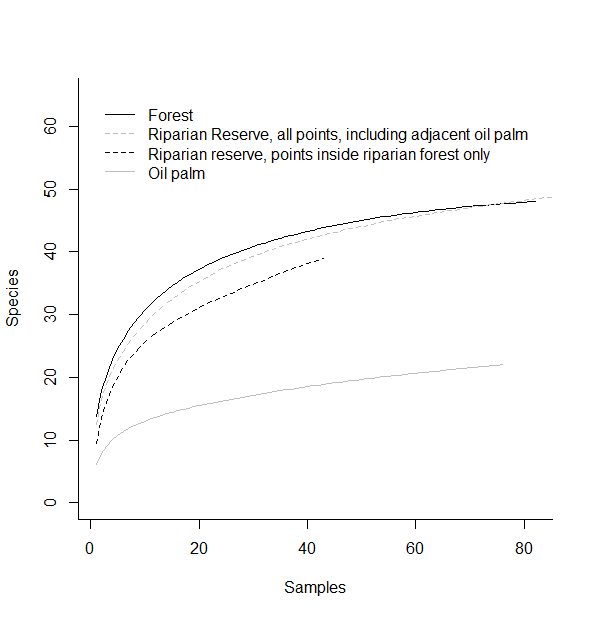


Fig S3. Species accumulation curves for data from baited pitfall traps.
